# Cardiovascular magnetic resonance: interstudy reproducibility of measurements of left ventricular function in children

**DOI:** 10.1186/1532-429X-13-S1-P359

**Published:** 2011-02-02

**Authors:** Jennifer A Bryant, Keith M Godfrey, Mark A Hanson, Sheila Barton, Charles R Peebles

**Affiliations:** 1 Southampton NIHR Nutrition, Diet & Lifestyle Biomedical Research Unit, Southampton, UK; 2 MRC Lifecourse Epidemiology Unit, Southampton, UK; 3 Southampton University Hospitals NHS Trust, Southampton, UK

## Introduction

In adults CMR is the gold standard technique for evaluation of LV function, with reportedly excellent interstudy reproducibility*.* In children compliance with breath-hold instructions and motion control can be significantly reduced and there is limited data on CMR reproducibility.

## Purpose

To determine the interstudy reproducibility of cardiovascular magnetic resonance (CMR) measurements of left ventricular (LV) end-diastolic volume (EDV), end-systolic volume (ESV), stroke volume (SV), ejection fraction (EF), cardiac output (CO), end-systolic mass (ESM) and end-diastolic mass (EDM) in children and examine the influence of analysis technique.

## Methods

CMR was performed in 10 healthy children aged 9 years as part of a study of developmental influences on cardiovascular structure and function. Contiguous short axis steady state free precession LV cine images were acquired. Scans were repeated following a short interval. Interstudy variability was assessed on datasets analysed using a manual post processing technique (Osirix). Each dataset was analysed with the papillary muscles and trabeculae included or excluded from the blood pool. Intra- and interobserver reproducibility was assessed on 10 datasets using both techniques.

## Results

Inter-study coefficients of variation (CVs) were similar for measurements including or excluding the papillary muscles and trabeculae, though marginally smaller for the latter. CVs for measurements excluding the papillary muscles and trabeculae were generally higher than those reported from studies of adults (CVs for EDV, ESV, SV, EF, CO, ESM and EDM were 8%, 10.9%, 9.7%, 4.4%, 10.5%, 10.7% and 8.6% respectively). Nonetheless, for all parameters the variability between subjects was greater than the interstudy and interobserver variability combined (e.g. for CO standard deviations were 1.83 for between subject variability and 0.42 for interstudy variability respectively).

## Conclusions

CMR measurements of cardiac structure tend to be less reproducible in children than in adults, probably due to difficulty in following breath hold instructions. Nonetheless, between subjects variability is greater than interstudy and interobserver variability, indicating that useful measurements can be obtained for research studies.

